# The cost of monitoring in time-based prospective memory

**DOI:** 10.1038/s41598-024-52501-w

**Published:** 2024-01-27

**Authors:** Gianvito Laera, Jasmin Brummer, Alexandra Hering, Matthias Kliegel, Sebastian Horn

**Affiliations:** 1 Cognitive Aging Lab (CAL), Faculty of Psychology and Educational Sciences, University of Geneva, 28 Boulevard du Pont d’Arve, 1205 Geneva, Switzerland; 2 Centre for the Interdisciplinary Study of Gerontology and Vulnerability, University of Geneva, Geneva, Switzerland; 3 LIVES, Overcoming Vulnerability: Life Course Perspective, Swiss National Centre of Competence in Research, Geneva, Switzerland; 4 Department of Psychology, University of Zürich, Zürich, Switzerland; 5 Department of Developmental Psychology, Tilburg School for Social and Behavioral Sciences, Tilburg University, Tilburg, The Netherlands

**Keywords:** Human behaviour, Psychology

## Abstract

Time-based prospective memory (TBPM) involves remembering to perform actions at specific times in the future. Several studies suggest that monetary consequences improve prospective remembering; however, the effect of monetary consequences on strategic time monitoring (i.e., clock-checking behaviour) in TBPM is still unknown. The present study investigated how the monetary costs on clock-checking affected TBPM accuracy and strategic time monitoring. Participants performed an ongoing lexical decision task while carrying out a TBPM task every two minutes. Motivational incentives were manipulated across three experimental conditions: a single-cost condition in which missed TBPM responses led to monetary deductions, a double-cost condition in which both missed responses and time monitoring led to monetary deductions, and a control condition with no monetary deductions. Overall, the findings indicated that monetary costs on clock-checking prompted more parsimonious strategic time monitoring behaviour, which negatively impacted TBPM accuracy. These results emphasize the importance of weighing the motivational aspects involved in strategic monitoring, shedding light on the complex relationship between clock-checking behaviour, its consequences, and TBPM performance.

## Introduction

How do we realize it is time to take a medication? Or to make our way to the train station to pick up a friend? These tasks involve prospective memory (PM): the ability to remember to execute intended actions at the appropriate moment in the future^1^. Time-based prospective memory (TBPM) refers to the ability to remember to perform an action at a specific time (e.g., picking up a friend at 10:00 from the station); event-based prospective memory (EBPM) refers to the ability to remember to perform an action in response to a specific event or situation (e.g., taking medication after dinner). There are several processes that contribute to successful PM performance, for example, monitoring time or using environmental events or activities as cues (e.g., always taking the medication in the kitchen).

TBPM is an integral part of many everyday activities, such as medication management, handling finances, or meal preparation^2–4^. In TBPM, time monitoring is needed to determine the correct time point for intention execution^5,6^. Yet, time monitoring can be costly: for instance, time monitoring can have detrimental consequences, as it imposes an additional cognitive task and requires attentional resources (e.g., while operating machinery, during conversation, when driving on the motorway, or during a medical procedure). Time monitoring may also have social costs (e.g., colleagues may perceive someone as impolite if they look at their wristwatch frequently during a meeting). The relation between the benefits of successful TBPM and the cost of time monitoring in real life is complex. This relation is influenced by our personal goals as well as the consequences of time monitoring behaviour^3,7^. Therefore, weighing the costs of monitoring (e.g., “Do I look in my calendar again during this meeting?”) against the benefits of successful remembering (“Will I catch my train?”) appears to be important. So far, however, we know very little about the relation between time monitoring, its consequences (in terms of cost), and TBPM. The present paper contributes to addressing this gap by investigating for the first time how a monetary cost of time monitoring affects TBPM (Note that we use the term *cost* here to refer to financial/economic and social costs, as well as to the effects that those costs have on attentional processes involved in PM. Therefore, the present conceptualization of cost is broader than in some of the previous PM studies, in which “cost” specifically refers to interference of the PM task with ongoing task performance).

## Importance and incentives effects

Previous research has found better prospective remembering for tasks that were considered as important^8,9^; such effects were also observed in naturalistic tasks and real-life environments, highlighting the role of perceived importance in TBPM and the relevance to study this aspect across different settings^10,11^. Perceived importance reflects subjective values of desired goals and expectations of anticipated consequences that can be manipulated by task instructions or incentives^12,13^. Penningroth and Scott^14^ have suggested that intentions that are related to personally relevant goals are perceived as more important and can improve performance in PM tasks through various cognitive processes, such as monitoring. Moreover, the importance of goals is predictive of attention allocation, monitoring, and strategy use^15^. So far, a few studies have examined if and how different monetary consequences influence PM in the laboratory^12,16,17^ and in naturalistic settings^10,11,18^. In a pioneering study by Meacham and Singer^19^, people were asked to mail post cards back to the researcher on pre-specified dates over a period of eight weeks. Participants in an incentive group, who received money for returning the cards on time, did so with higher probability and more of them indicated the use of external reminders (e.g., calendars) to support their PM than participants in a no-incentive group. This suggests that monetary consequences may increase the perceived importance of a PM task and induce the use of mnemonic strategies. Relatedly, Horn and Freund^13,17^ compared the effect of monetary gain incentives (for accurate responses on PM target events) and loss incentives (deductions from a monetary endowment for missed PM responses) in EBPM across adulthood. Results indicated that both gain- and loss-incentives (compared to a control condition without performance-contingent incentives), improved PM accuracy, even though individual differences (e.g., age differences^20^) appear to play an important role. These and other studies suggest that monetary consequences affect PM accuracy^12,13,16–18^. To our knowledge, however, the impact of incentives on monitoring has not been investigated. As argued above, a better understanding of the cost of time monitoring is particularly relevant in TBPM tasks, as many scenarios come with secondary monitoring costs. Therefore, one goal in the present study was to systematically investigate the cost of time monitoring in TBPM.

## The cost of time monitoring

For a given TBPM task, time monitoring can be measured as the mean (or sum) of clock checks, regardless of when these clock checks are made (absolute clock checking), or as the mean of clock checks over specific intervals of time. Time monitoring is called *strategic* if its pattern follows a J-shaped curve, with few checks in the initial phases of a TBPM task (when the target time is further away), followed by an exponential growth of clock checks as the relevant target time approaches^5,6,21–24^. Both absolute frequency and strategy use of time monitoring tend to correlate positively with TBPM accuracy^5,21,23–25^.

In the majority of TBPM studies, time monitoring has been unconstrained and self-paced, meaning that participants could check a clock whenever they wished, without imposing any cost. Only few studies have used experimental designs with restrictions on time monitoring. For example, Harris and Wilkins^26^ placed the clock behind participants’ backs, requiring an overt turning around^27^. Huang et al.^28^ instructed participants to use the timer as infrequently as possible in one of their experimental conditions, whereas Mioni and Stablum^25^ permitted participants in one experimental condition to check the clock only up to six times over the course of five minutes. Overall, these studies suggest that when restrictions were imposed on time monitoring in younger adults, the frequency of clock-checks decreased, but strategic time monitoring behaviour increased^25,26,28^. However, in all these studies, constraints on time monitoring were imposed by experimenters with instructions; yet, in daily life, people might instead self-regulate their clock-checking behaviour according to their personal goals, their perceived task importance, or the consequences related to task fulfilment (based on individual preferences and utility). Thus, it is important to understand the interplay between motivation and cognition in the context of TBPM, that is, how participants modulate processes underlying strategic time monitoring and TBPM performance when TBPM is motivated by higher levels goals and individual preferences.

## The present study

The “down-stream” effects (i.e., the influence of high-level goals on lower-level cognitive processes, such as attention, perception, and memory) of costs of time monitoring and TBPM failures are currently unknown^29^. This appears surprising, given the importance that these effects can have in daily life (e.g., forgetting to take medication can lead to serious health issues; forgetting to pay bills on time can lead to extra fees). Moreover, it is unknown if and how motivational incentives affect the relation between time monitoring and TBPM^16,30^. To fill this gap, the present study investigated the effect of monetary costs on time monitoring and TBPM. Participants made ongoing lexical decisions and were additionally asked to press the ENTER-key every two minutes as TBPM task (while having the possibility to check a clock whenever they wished). We manipulated motivation to monitor time in three experimental conditions: in one group of participants, missed PM responses were penalized with a monetary deduction from an initial endowment of £6 (single-cost condition). In a second experimental group (double-cost condition), not only missed PM responses, but also time monitoring resulted in deductions from the endowment. Lastly, in a control group, participants received no information regarding an additional incentive prior to the experiment. We measured TBPM accuracy as mean proportion of correct PM responses within an interval (± 6 s) around the PM target time (every 2 min); for time monitoring, we measured mean frequency of clock checks over time and absolute and relative clock checks^23,31^. Relative clock-checking is a quantitative index of strategic behaviour based on the tendency to concentrate clock checks in the last interval before a specific PM target time; a strength of this measure is that relative clock-checking accounts for individual differences in TBPM that are often considered as noise in more traditional analyses of time monitoring^5,25,32^; for further information, see Joly-Burra et al.^31^.

**Figure 1:**
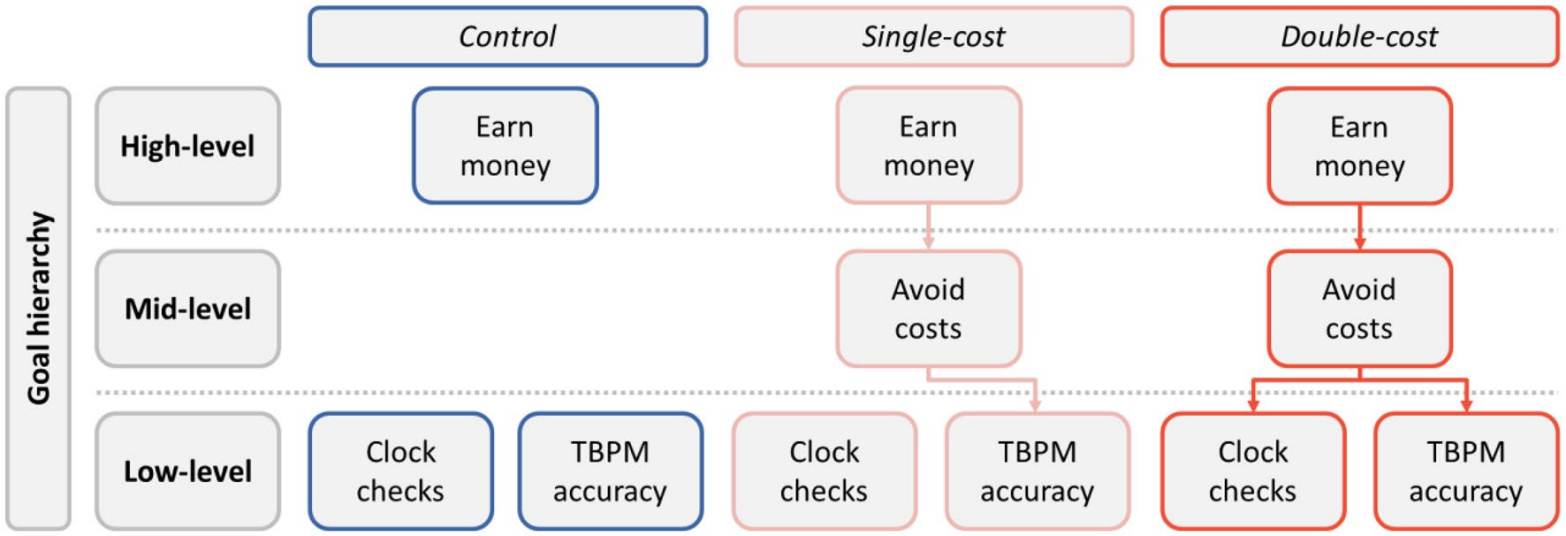
Cognitive-motivational interplay as a function of experimental conditions. *TBPM* time-based prospective memory. The figure represents how goal hierarchy (low-, mid-, and high-level) affects behavioral performance (clock checks and accuracy) and motivational tendencies (earn money and avoid costs) in a time-based prospective memory task across experimental conditions (control, single-, and double-loss condition). Arrows indicate direct downstream effects of experimental conditions (i.e., single- and double-cost) on goal hierarchy and TBPM performance (as both clock-checking and accuracy). In all conditions, participants aimed to earn money (i.e., the high-level goal). In the control condition, the high-level goal didn't impact directly the low-level goal of performing the prospective memory task accurately, because such behavioral performance was not tied to any external incentives. In the single-cost condition, the opportunity to earn extra payment through better performance (i.e., avoiding loss of points later converted in money) added a mid-level motivation that was selectively related to the accuracy in the TBPM task; in the double-cost condition, clock checks were also penalized with a monetary loss, adding further motivation to avoid losses.

Based on research on motivated cognition^13–15,33^, we considered three different scenarios for the different conditions, as depicted in Fig. 1. According to our theoretical considerations, in the control condition, the TBPM task was only linked to a very general and abstract “higher-level” goal (i.e., get remuneration for participation in the study); by contrast, in the single-cost condition, TBPM accuracy was directly associated with additional monetary consequences, and might be linked with a more concrete “mid-level” goal (i.e., “avoid monetary losses in this task”) which, in turn, could be linked to a more generic “higher-level” goal of getting money for participation. Finally, in the double-cost condition, both TBPM accuracy and time monitoring might be directly linked with a more concrete “mid-level goal”. In Fig. 1, arrows entering the boxes symbolize the direct impact of mid-level goals on the two primary behaviours observed in TBPM tasks, namely clock-checking and accuracy. Therefore, the absence of an arrow into clock-checking in the single-cost condition indicates that clock-checking was not directly associated with any mid-level goal. Consequently, changes in clock-checking in this condition were primarily thought to be influenced by costs directly connected to TBPM accuracy. In contrast, in the double-cost condition, it is assumed that clock-checking is directly linked to the mid-level goal of avoiding additional monetary costs. Consequently, variations in clock-checking in this condition are driven by costs linked to both clock-checking and TBPM accuracy.

Based on these ideas, we expected that participants in the double-cost condition would use the clock less frequently, but more strategically, than participants in the single-cost or control condition, as only in the double-cost condition, time monitoring would be linked with a “mid-level” goal of avoiding cost. We also expected that strategic behaviour in time monitoring correlates positively with TBPM accuracy, especially in the double-cost condition^5,6,21,24,31^. We further expected that TBPM accuracy is highest in the single-cost condition because there was a motivational incentive to avoid losses (following PM misses) while having the opportunity to engage in time monitoring “free of charge”. In contrast, participants in the double-cost condition were expected to check the clock more parsimoniously to avoid further costs, which might decrease TBPM accuracy compared to the other conditions.

## Methods

### Participants

This study was statistically powered to detect small-to-medium differences in performance between experimental conditions (control versus single-cost versus double-cost). We computed required sample size a priori, using the Software G*Power^34^; given that no previous study investigated the cost related to TBPM and/or time monitoring, we calculated the power analysis establishing an a-priori effect size of *d* = 0.20. The power analysis indicated that detecting an effect size of 0.20 at 95% power (two-tailed test, α = 0.05), would require a sample of a total of 102 participants in an ANOVA test with 3 independent groups (i.e., control, single-cost, and double-cost condition) and two repeated measures (i.e., two task blocks: ongoing task baseline and TBPM block). To increase the statistical power, we recruited a total of 210 participants, hence doubling the sample size obtained from the power analysis, as suggested from previous online studies^35,36^; all participants were recruited via the online research provider Prolific.co, using the following inclusion criteria: age between 18 and 35 years, fluent in English, and following exclusion criteria no current alcohol therapy, no head injury, no long-term health condition/disability, no chronic condition/illness, no mild cognitive impairment/dementia, no mental illness/condition, and no medication intake. All participants provided informed consent before participating in the study. The study was performed in accordance with the Declaration of Helsinki. The study protocol had been approved by the ethics commission of the University of Geneva (CUREG-2022-11-122). Participation was reimbursed with a fixed hourly compensation of £9.00. In addition, all participants could obtain a performance-contingent bonus of up to £6.00.

### Materials

All tasks were programmed in PsychoPy, version 2021.2.3^37^ and hosted on Pavlovia (https://pavlovia.org/^38^). All materials are available in the repository of the Open Science Framework (10.17605/OSF.IO/V5W8X).

#### Tasks

Participants performed two task blocks: one ongoing task block without a TBPM task and one block with an embedded TBPM task. In both blocks, the ongoing task was a lexical decision task (LDT), in which stimuli were letter strings; participants were asked to respond whether a string formed an English word or not. All stimuli were taken from the English Lexicon Project database^39^. In total, 306 stimuli (153 words) were selected based on average scores of standardized accuracy, frequency (only for words), and response times; all stimuli (words and non-words) had between 5 and 8 letters. Each ongoing task trial started with a fixation cross (1 s), followed by the stimulus (2 s), and a subsequent blank period (black screen) that lasted randomly between 1 and 2 s. All ongoing task stimuli were presented in fully randomized order across the blocks. In total, 153 ongoing task trials were included within each block; the average duration of each block was ~ 10.20 min. During the TBPM task, participants were asked to remember to press the ENTER key on the keyboard every 2 min while performing the LDT; in total, five PM responses were collected for each block; moreover, participants were free to check the clock as often as they wanted by pressing the SPACEBAR; if they did so, a digital clock (format: "00:00") appeared on the computer screen for 3 s.

#### Further questionnaires

After participants completed the TBPM task, they were asked to indicate which task was more important for them (ongoing task, TBPM task, or both; 77% declared both tasks were equally important, 17% declared that the TBPM task was the most important task, and 6% declared that ongoing task was the most important task; exploratory analyses on subjective task importance are reported in the Supplementary Materials). Other collected measures, which are beyond the scope of the present paper, were the following: Participants indicated how they perceived time during the ongoing task baseline and the TBPM task^40^, responded to a scale of loss aversion^41^, a scale of time experience^42^, and a follow-up questionnaire to indicate whether any strategy to track the passage of time during the TBPM task had been used; specifically, participants were asked to give binary responses (yes/no) to this question, and in the case of a yes response, were asked to provide a brief explanation. Sociodemographic data were obtained from prolific.

### Procedure

Overall, a session lasted ca. 30–35 min. Prior to participation, all relevant information concerning the experimental procedure and data access were provided in written form on the screen; participants provided informed consent before. Participants then read instructions for the ongoing task baseline block. However, before moving to the practice block, they went through an instruction quiz (i.e., participants had to answer correctly to all questions about task instructions before proceeding^35^). If participants passed this attention check, they performed a short practice session of the ongoing task baseline (comprising 8 trials, 4 words and 4 nonwords). Once participants reached an ongoing task accuracy of at least 75%, they moved on to the ongoing task baseline block, otherwise they repeated the practice block. After that, participants read instructions for the TBPM task and were randomly assigned to one of three conditions: in the single-cost and double-cost conditions, participants were informed that they would now receive an initial endowment of + 100 points before performing the TBPM task. In the single-cost condition, participants were then informed that any missed PM response (i.e., missing to press ENTER during the target-time window) led to a loss of − 10 points, deducted from the initial endowment of 100 points; in the double-cost condition, participants were additionally informed that time monitoring also had a cost and led to a loss of − 2.5 points each time they pressed the SPACEBAR to check the clock. Participants were also told that the final points score would later be converted into a monetary bonus. However, the specific conversion rate was not stated, allowing us to adjust for any disadvantage in points retained, due to the experimental condition. In the control condition, participants were simply instructed to perform the TBPM task, without explicit mentioning of any incentives (participants in the control condition received the bonus payment as well, in order to ensure equality of remuneration across groups; however, they were informed about it only after they completed the TBPM task). Participants performed another instruction quiz about the TBPM task; if participants responded correctly to all the questions, another practice block was administered (lasting approx. 2 min), allowing participants to familiarize themselves with the TBPM task. If participants correctly performed the PM response and reached an ongoing task accuracy of at least 75%, the TBPM block started. Following this, participants responded to follow-up questionnaires and were debriefed.

## Results

Data pre-processing and figures were carried out in *R* version 4.2.1^43^ with the support of ChatGPT for building *R*-scripts^44^. The analyses were carried out in Jamovi, version 2.3.21.0^45^. We excluded three participants from the analyses due to technical computer problems causing bad data quality and/or missing data; one further participant was excluded due to failure of understanding the PM task. In total, 206 participants were included in the final analyses; descriptive statistics of TBPM accuracy and time monitoring are in Table 1. The analyses are reported in two parts: first, ANOVAs were calculated to investigate the effects of the experimental manipulations on TBPM accuracy and time monitoring. Second, a multi-group path analysis was carried out to explore the strength of the predictive association between time monitoring and TBPM performance across experimental conditions. For all ANOVA analyses, the effect sizes were calculated using partial eta squared values (η2_*p*_). Post-hoc *t*-tests were carried out applying Bonferroni’s correction to the *p*-values (indicated as *p*_*adj*_). For all statistical analyses, the alpha level was set at 0.05.

**Table 1:** Descriptive statistics.

Experimental condition	TBPM accuracy	Monitoring over time				Absolute clock-c	Relative clock-c. (%)
		t1	t2	t3	t4		
*M*							
Control	0.91	0.78	1.22	1.53	2.87	32.03	45.98
Single-cost	0.95	0.79	1.18	1.47	2.97	32.03	49.64
Double-cost	0.84	0.34	0.47	0.67	1.43	14.59	58.00
*SD*							
Control	0.16	0.72	0.76	0.83	1.16	15.35	12.42
Single-cost	0.10	0.72	0.88	0.83	1.12	15.48	14.45
Double-cost	0.22	0.58	0.72	0.79	1.34	16.13	25.56

Descriptive statistics of time-based prospective memory accuracy, mean monitoring over time, as well as absolute and relative clock-checking, as a function of the experimental conditions (monetary loss: control, single-loss, double-loss). *TBPM* time-based prospective memory, *t1* time 1 (i.e.: first 30 s’ interval before the PM target time), *t2* time 2 (i.e.: second 30 s’ interval before the PM target time), *t3* time 3 (i.e.: third 30 s’ interval before the PM target time), *t4* time 4 (i.e.: fourth and last 30 s’ interval before the PM target time), *clock-c.* clock-checking.

### Time-based prospective memory

**Figure 2:**
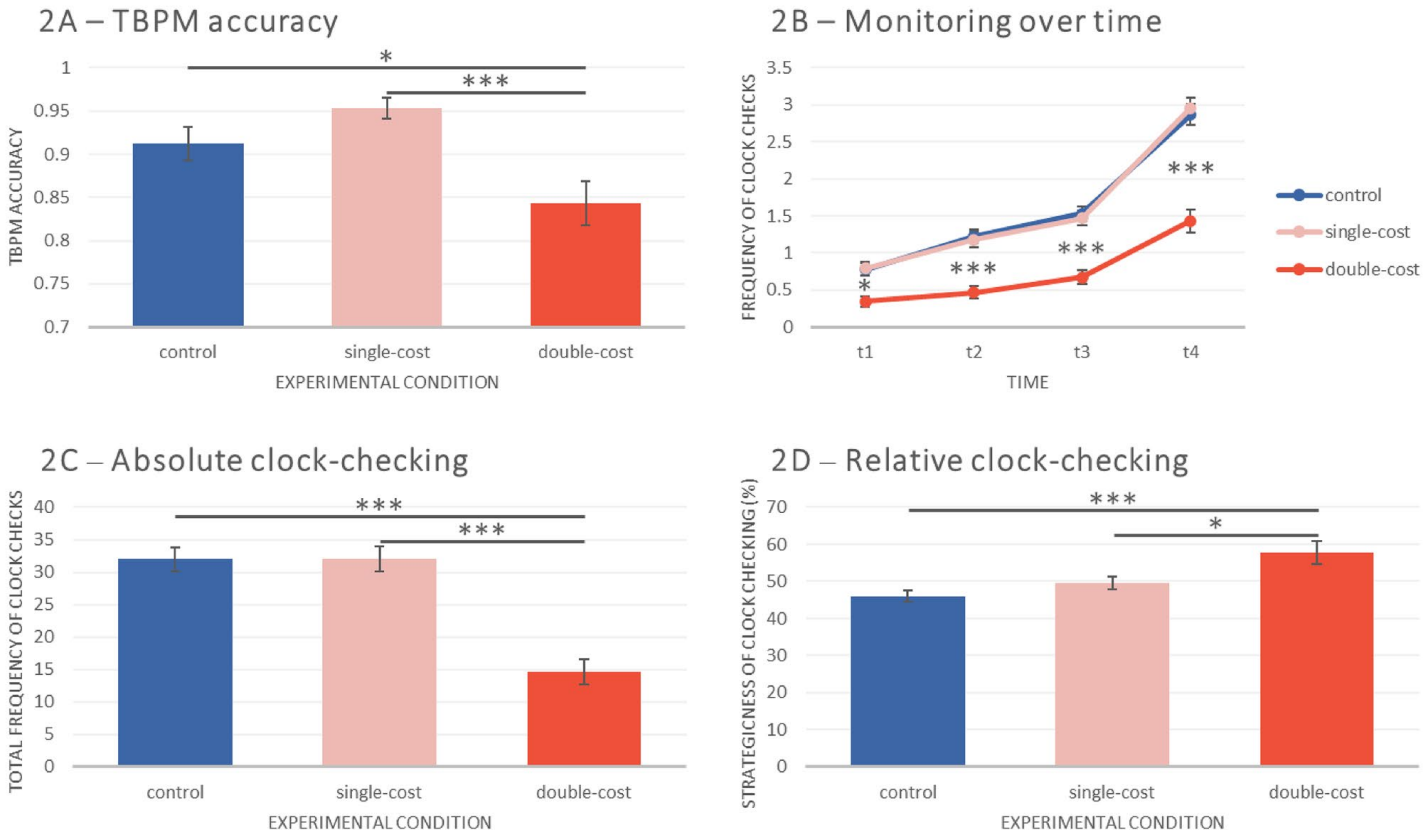
Main results from ANOVAs. The figure shows accuracy in the time-based prospective memory task (**A**), the mean frequency of clock checks over time (**B**), as well as absolute (**C**) and relative clock-checking (as a percentage; **D**) as a function of the experimental conditions (control, single-cost, double-cost). Error bars indicate standard error of the mean. *TBPM* time-based prospective memory, *t1* time 1 (i.e., first 30 s interval before the PM target time), *t2* time 2 (i.e., second 30 s interval before the PM target time), *t3* time 3 (i.e., third 30 s interval before the PM target time), *t4* time 4 (i.e., fourth and last 30 s interval before the PM target time). *p < 0.05; **p < 0.01; ***p < 0.001.

TBPM accuracy (see Fig. 2A) was measured as mean proportion of correct PM responses. A PM response was considered correct if it was made within ± 6 s around PM target time (equivalent to 10% of the total interval of 2 min between PM target times^24^). The main effect of the experimental manipulation (control vs. single-cost vs. double-cost) was significant, indicating that TBPM accuracy differed between conditions, *F*(2, 203) = 7.82, *p* < 0.001, η^2^_*p*_ = 0.072. Post-hoc Bonferroni comparisons indicated that participants in the double-cost condition had lower TBPM accuracy than participants in the control condition, *t*(203) = − 2.45, *p*_*adj*_ = 0.046, and than participants in the single-cost condition, *t*(203) = 3.91, *p*_*adj*_ < 0.001.

### Time monitoring

For the analysis on time monitoring, we carried out a series of ANOVAs. We aimed to investigate to what extent participants were strategic when checking the clock^31^ and whether the experimental cost manipulation affected the frequency of clock checks over time. We calculated a 3 × 4 mixed ANOVA with between-subjects factor *Condition* (control vs. single-cost vs. double-cost) and within-subjects factor *Time* (*t*_1_ to *t*_4_) to analyse time monitoring (frequency of clock checks). The factor Time refers to four time-intervals of 30 s each (i.e., *t*_1_ represents the first 30 s before a PM target time; *t*_2_ represents the second 30 s; *t*_3_ represents the third 30 s; *t*_4_ the final 30 s before the target time). Afterwards, two further one-way ANOVAs were carried out on absolute and relative clock-checking, shown in Fig. 2C and D. Absolute clock-checking was calculated as the sum of clock checks over five PM target windows (i.e., total frequency of clock checks over the entire TBPM task block). Relative time monitoring was computed starting from the frequency of clock checks over four intervals of 30 s (*t*_1_ to *t*_4_, as in the above analysis), considered separately for each of the five TBPM tasks; then, for each TBPM task, the number of clock checks during the last time interval (*t*_4_) was divided by the total number of clock checks (i.e., from *t*_1_ to *t*_4_), and averaged across the five TBPM tasks for each participant, as follows:

1 $$Relative \, clock-checking = \left(\frac{{\sum }_{i=1}^{5 }\frac{{t4}_{i}}{{t1}_{i}+{t2}_{i}{+t3}_{i+}{t4}_{i}}}{5}\right)*100$$

where *t* = time-window, and *i* = index of the current PM task. This index is a percentage score ranging from 0 to 100%, with 100% representing the highest strategic behavior possible (i.e., all clock checks made in the last interval before the PM target time^31^).

The results of the analysis on the frequency of clock checks over time showed a significant main effect of Time, *F*(2.02, 410.65) = 500.17, *p* < 0.001, η^2^_*p*_ = 0.71, a main effect of Condition, *F*(2, 203) = 28.67, *p* < 0.001, η^2^_*p*_ = 0.22, and a Time × Condition interaction, *F*(2.02, 410.65) = 18.14, *p* < 0.001, η^2^_*p*_ = 0.15. Post-hoc comparisons for the main effect of Time revealed that participants strategically monitored the clock, indicating less frequent clock checks during *t*_1_ than *t*_2_, *t*(203) = − 9.40, *p*_*adj*_ < 0.001, than *t*_3_, *t*(203) = − 14.74, *p*_*adj*_ < 0.001, and than *t*_4_, *t*(203) = − 27.43, *p*_*adj*_ < 0.001. Similarly, participants checked the clock less frequently in *t*_2_ than *t*_3_, *t*(198) = − 7.39, *p*_*adj*_ < 0.001, and than *t*_4_, *t*(198) = − 25.28, *p*_*adj*_ < 0.001. Clock-checking frequency was also lower in *t*_3_ than *t*_4_, *t*(198) = − 22.15, *p*_*adj*_ < 0.001. Post-hoc comparisons for the Time × Condition interaction showed that participants in the double-cost condition checked the clock less frequently than participants in both the control and single-cost conditions in a consistent manner over time, whereas the pattern of monitoring did not differ between control and single-cost conditions (note the overlapping lines in Fig. 2B). Post-hoc comparisons between experimental conditions indicated that participants in the double-cost condition monitored time less frequently than participants in the control condition, *t*(198) = − 6.52, *p*_*adj*_ < 0.001, and than participants in the single-cost condition, *t*(198) = − 6.54, *p*_*adj*_ < 0.001. The difference in time monitoring between control and single-cost condition was not significant (*p*_*adj*_ = 1). These results were mirrored by the analysis of absolute clock-checking (Fig. 2C), showing a main effect of the experimental manipulation, *F*(2, 203) = 28.67, *p* < 0.001, η^2^_*p*_ = 0.22. The analysis of relative clock-checking (Fig. 2D) also showed a significant effect of condition, *F*(2, 197) = 7.49, *p* < 0.001, η^2^_p_ = 0.07. Notably, however, post-hoc comparisons revealed that the participants in the double-cost condition were more strategic than participants in the single-cost condition, *t*(203) = 2.63, *p*_*adj*_ = 0.027, and than participants in the control condition, *t*(203) = 3.79, *p*_*adj*_ < 0.001; the pairwise comparison between control and single-cost condition was non-significant (*p*_*adj*_ = 0.728). Thus, a reversed pattern emerged for absolute and strategic/relative clock checking.

**Figure 3:**
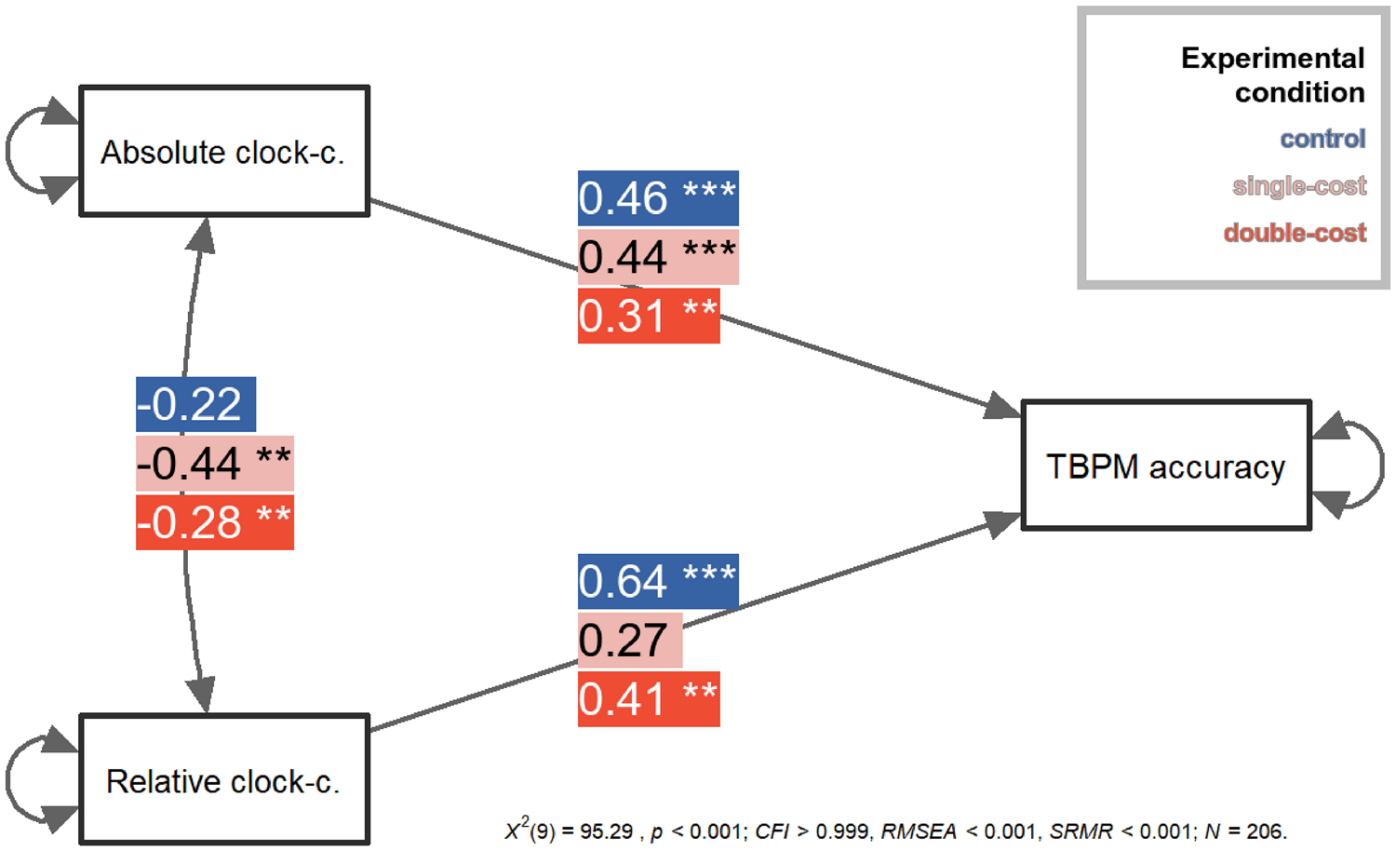
Results from multi-group path analysis. A graphical representation of the model tested in the path analysis, with regression and covariance coefficients for each experimental condition (monetary loss: control, single-loss, double-loss). *TBPM* time-based prospective memory, *clock-c*. clock-checking. **p < 0.01; ***p < 0.001.

THj in Jamovi^46^ to shed light on the relation between different aspects of time monitoring, TBPM accuracy, and type of cost/incentive. The tested path model (see Fig. 3) comprised both absolute and relative clock-checking as predictors of TBPM accuracy across experimental conditions (control vs. single-cost vs. double-cost). The two monitoring measures were allowed to correlate. A robust maximum-likelihood algorithm was used for model estimation; adjusted bias-corrected bootstrapping with 1000 samples was performed to calculate standard errors (for further statistical details, see the Supplementary Materials).

The multi-group model with no equality constraints provided a good statistical fit to the data, χ^2^(9) = 95.29, *p* < 0.001, *CFI* > 0.99, *RMSEA* < 0.001, *SRMR* < 0.001 and explained a significant portion of variance in TBPM accuracy for the control group (*R*^2^ = 0.49, χ^2^_Wald_(2) = 15.14, *p* < 0.001), for the single-cost condition (*R*^2^ = 0.16, χ^2^_Wald_(2) = 12.74, *p* = 0.004) and for the double-cost condition (*R*^2^ = 0.19, χ^2^_Wald_(2) = 10.92, *p* = 0.004). Constraining the regression coefficients in the model to be equal across the three subgroups resulted in a statistically significant misfit (χ^2^(4) = 19.50, *p* < 0.001, *CFI* = 0.82, *RMSEA* = 0.089, *SRMR* = 0.241), suggesting that effects of time monitoring on TBPM accuracy were different across experimental conditions. Specifically, the model showed that both absolute and relative clock-checking predicted TBPM accuracy in the double-cost condition; however, relative clock-checking better predicted TBPM accuracy (β = 0.41, *p* = 0.004) than absolute clock-checking (β = 0.31, *p* = 0.012). The same pattern was found in the control condition (β_relative_ = 0.64, *p*_*relative*_ < 0.001; β_absolute_ = 0.46, *p*_*absolute*_ < 0.001). By contrast, only absolute clock-checking predicted TBPM accuracy in the single-cost condition (β = 0.44, *p* < 0.001), whereas the effect of relative clock-checking was not significant (*p* = 0.101). Absolute and relative clock-checking also correlated with each other in the single-cost condition (β = − 0.44, *p* = 0.002) and double-cost condition (β = − 0.28, *p* = 0.004), but not in the control group (*p* = 0.151). Hence, we further assessed whether the relationship between the two monitoring measures was the same across experimental conditions by testing a further constrained model, in which the covariance between absolute and relative clock-checking was constrained to be equal across experimental conditions. The results showed that constraining covariances to be equal across the three subgroups did not lead to significant misfit, χ^2^(2) = 3.15, *p* = 0.207, *CFI* = 0.98, *RMSEA* = 0.093, *SRMR* = 0.058, suggesting that the relationship between monitoring measures was similar across experimental conditions (however, some error was introduced in the new constrained model, as *RMSEA* = 0.093).

## Discussion

PM tasks that are perceived as important are usually remembered more likely than less important ones^8,9,47^. Monetary incentives increase the importance of PM tasks^12,13,19^, but the effects on monitoring strategies are hardly understood. The present study addressed the novel question of how monetary costs affect monitoring and accuracy in TBPM. In one group of participants (single-cost condition), missed PM responses were penalized with a monetary deduction from an initial endowment of £6. In a second group (double-cost condition), missed PM targets as well as clock checks were associated with a cost and resulted in deductions from an initial endowment of £6. In a control group, participants received no information regarding an additional incentive prior to the experiment.

Based on previous research on motivated cognition^14,15,17,33^, we expected that participants in the double-cost condition use the clock less frequently, but more strategically, than participants in the single-cost or control condition; we also expected that strategic behaviour in time monitoring correlates positively with TBPM accuracy, especially in the double-cost condition^5,6,21,24,31^. The findings are largely in line with these expectations: even though participants generally tended to increase clock checks over time before a target time approached (Fig. 2B), overall clock checks were substantially lower in the double-cost than the other conditions (Fig. 2C). At the same time, participants in the double-cost condition showed the highest rate of strategy use (Fig. 2D), despite the lower number of overall clock checks. Monitoring patterns over time, absolute, and relative clock checking did not differ between the single-cost and control conditions, indicating that clock-checking was only affected when time monitoring was directly charged. Both absolute and relative clock-checking correlated positively with TBPM accuracy, suggesting that providing motivational incentives just for TBPM accuracy did not affect monitoring; in the single-cost condition, the effect of relative clock checking on TBPM accuracy even failed to reach significance (Fig. 3).

Regarding TBPM accuracy, we expected highest performance in the single-cost condition, because there was a motivational incentive to avoid losses (following PM misses) and simultaneous opportunity for time monitoring that was “free of charge”. In contrast, we expected participants in the double-cost condition to be more cautious about checking the clock to avoid an extra cost, which might decrease TBPM accuracy compared to the other conditions. Indeed, TBPM accuracy was highest in the single-cost condition and lowest in the double-cost condition (Fig. 2A), with a significant difference in performance between these two conditions, as well as between double-cost and control condition. Exploratory analyses of ongoing task performance only revealed that, in both task blocks (ongoing task baseline and TBPM), participants in the double-cost condition performed more accurately than participants in the other two conditions (the same comparison between control and single-cost condition was not significant) but all other effects on accuracy were not significant; moreover, there were no effects of conditions on ongoing task response times (see Supplementary Materials for further information). This suggests that the differences in time monitoring observed between the conditions are unlikely due to trade-offs with ongoing task performance.

### The interplay between monetary cost and monitoring in TBPM

Overall, our results are in line with a set of PM studies, which indicated that, when restrictions or costs are imposed on time monitoring in younger adults, the frequency of clock-checks decreased, but strategic behaviour increased^25,26,28^. However, in contrast with these previous studies that imposed constraints via task instructions, the effects on clock checking in the present study reflect preferential behaviour, self-regulated by participants based on perceived utility; we did not impose any instructional constraints on how to check the clock. Furthermore, and in contrast to Huang et al.’s conclusion that clock-checking constraints do not impact TBPM accuracy^28^, our results indicated a small yet significant effect. These differences across studies may be attributed to variations in the experimental manipulations. Future studies are needed to compare instructionally driven and incentive-driven constraints, and how these constraints affect strategic time monitoring and TBPM performance.

Considering that monetary incentives may affect attention allocation and strategy use on relatively lower levels of a goal hierarchy^15,33^, the behavioral pattern of clock checking can also help to better understand how motivational incentives affect cognitive processes in time monitoring. Specifically, it is possible that, when clock-checking was costly (i.e., in the double-cost condition), participants might have engaged internal time mechanisms^5,48^, especially when the PM target time was expected to not occur soon, allowing participants to concentrate the majority of the overall clock checks temporally closer to the PM target time^28^ (Fig. 2D). In contrast, if monitoring was not costly, the strategic involvement of internal time-estimation processes might have been reduced, especially in the single-cost condition, in which the strategic use of time monitoring did not significantly predict TBPM accuracy (Fig. 3); this pattern suggests that the tendency of being strategic was not essential for good performance when misses in the TBPM task came with a cost, but clock checking was “free of charge”. However, these speculations need to be investigated in future studies, as the relationship between internal time-estimation processes and relative/absolute monitoring is unknown. Interestingly, the modulation of strategic/relative monitoring as a function of the experimental manipulation (Fig. 2D) was found despite any change in the monitoring “J-shaped” curve over time as a function of the experimental manipulation (Fig. 2B); such apparent discrepancy is due to the fact that analysing monitoring over time is based only on absolute frequency of clock-checking, which cannot capture strategic monitoring on a trial-by-trial basis. In contrast, absolute and relative clock-checking scores are calculated trial-by-trial and, and such, do account for individual differences that are neglected in the traditional analysis of the mean frequency of clock checks over time. Therefore, the present findings also support Joly-Burra et al.’s notion that using relative clock-checking in addition to absolute clock-checking is more informative and provided important additional information above and beyond the mean frequency of monitoring over time^31^.

Monetary cost had clear effects on time monitoring, but monetary-incentive effects on TBPM accuracy were more subtle, even though the pattern was in line with our expectations. This might be due to the specific combination of a monetary cost for clock checks and misses in the TBPM task. Considering that TBPM correlated positively with absolute clock-checking across experimental conditions (Fig. 3), it appears reasonable to argue that this pattern reflects the differential effects of motivational incentive(s) on time monitoring. The results might suggest that participants achieved fairly high accuracy (> 85%), but through the use of different time-monitoring strategies across conditions: in the double-cost condition, time monitoring was linked with a “mid-level goal”, whereas this was not the case in the single-cost and control conditions; indeed, monitoring behaviour was very similar between these two latter conditions, whereas participants in the double-cost condition exhibited a more strategic monitoring behaviour.

Another interesting point is that the present study focused on the “downstream effect” of monetary incentives on TBPM performance (i.e., the effects of monetary incentives on attention and monitoring on a concrete task level). However, it will be relevant in the future to examine the role of “up-stream effects”, too (e.g., if and how specific features of the memory tasks moderate the motivational influence from a higher-level goal, such as making money). For example, the difficulty of the TBPM task, the number of intentions^49,50^, or possibilities to check the clock^25,28^ can modulate the allocation of attention and strategy use in TBPM and possibly affect goals on “higher levels” of a goal hierarchy^14,15,33^. Future studies are needed to further explore the interplay of such up-stream effects and motivational incentives in PM.

## Limitations and outlook

The present experiment was conducted online. Some studies suggest that performance is comparable across laboratory and online settings^35,51–53^, also in the domain of PM^54,55^. We included several attention checks in our study; only participants who passed these checks were included in our analyses. Nonetheless, a replication in the laboratory would be useful to account for potentially higher levels of noise in the data from online studies^53,56^. Moreover, TBPM accuracy was generally high, suggesting that the task used in this study was easy for participants. It is possible that effects of motivational incentives (e.g., differences between control and incentivized conditions) are better detected if accuracy is lower and variability in TBPM is higher. The relatively high TBPM accuracy might have obscured potential effects between the control and single-cost condition. Therefore, it will be interesting to investigate incentive effects with tasks that vary in difficulty/demands. Moreover, the use of monetary incentives may not well capture the consequences and types of cost encountered in some daily-life settings. For instance, consequences of PM misses (e.g., missing an important deadline or appointment), could have different motivational and cognitive implications in daily life. It will be interesting to investigate these issues in naturalistic settings as well^19^. Another limitation is that, in the design of this study, there was no condition in which only time monitoring was penalized with monetary deductions (without charging the TBPM task); we decided for such a design because having another single-cost condition in which only time monitoring was charged could have potentially incentivized participants to engage in cheating behaviour or even neglect the TBPM task altogether. Future laboratory studies are needed to replicate our findings and to possibly consider single-cost conditions, in which only time monitoring is charged with monetary deduction. Finally, it will be important to systematically compare the perceived cost of monitoring and the benefit of successful remembering, and how such costs and gains are integrated.

Overall, the present findings show for the first time that monetary costs affect time monitoring as well as accuracy in TBPM. If the cost of time monitoring is high, people check their clocks more strategically and parsimoniously. However, more parsimonious monitoring can detrimentally affect prospective remembering (as in the present study). This highlights the importance of carefully weighing the costs of monitoring (e.g., “Do I look at my watch again?”) against the benefits of successful remembering (“Do I leave the meeting now to catch my train?”). The present study can also corroborate findings obtained from naturalistic settings. For instance, some studies investigated the effect of incentives or perceived task importance in PM tasks carried out within naturalistic settings, showing that perceived importance can explain, at least partially, a significant portion of both within- and between-subjects variance in the likelihood of successful intention completion in daily life^10,11,18,19^. Given the ubiquitous presence of TBPM tasks in daily life^10,11^, it is important to understand when a PM task is likely to be fulfilled, and when and for whom it is likely to fail, including how consequences related to personal goals affect strategic time monitoring and TBPM performance.

### Supplementary Information

Supplementary Information.

## Data Availability

Data are available in the repository of Open Science Framework, along with R-codes and statistical results (10.17605/OSF.IO/V5W8X).
