# Supplementary material for: The cost of monitoring in time-based prospective memory
Source: Sci Rep. 2024 Jan 27;14:2279. doi: 10.1038/s41598-024-52501-w (PMC10821954; doi:10.1038/s41598-024-52501-w)
Supplement: Supplementary file 1 — Supplementary Information. [file 41598_2024_52501_MOESM1_ESM.docx]

The Cost of Monitoring in Time-Based Prospective Memory

# SUPPLEMENTARY MATERIAL

In the present document, all the details of the statistical analyses were reported, along with relevant references and formulas. Furthermore, we reported exploratory analyses on ongoing task (OT) performance and subjective task importance.

# Statistical analyses

In this sections, further details of statistical analyses are reported along with additional – exploratory – analyses on OT performance and subjective task importance. In Figure 1, the main results from the manuscript are depicted using raincloud plots for transparent data visualization.

## Path analyses

For the multi-group path analysis, *R*^2^ was computed as one minus the standardized residual variance of the endogenous variable (i.e., TBPM accuracy); moreover, the confidence intervals were estimated by converting *R^2^* to *R*, then to *z*-scores using the Fisher Z-transformation; afterwards, the confidence interval for *z*-scores were estimated^1^, and then back transformed them to *R^2^*. The inferential tests associated with the *R^2^*’s were obtained using the Wald’s chi-squared tests (χ^2^_Wald_) comparing the original model with a constrained model in which regression coefficients leading to the endogenous variable (i.e., TBPM accuracy) were set to zero (except the intercept). The Comparative Fit Index (*CFI*), the Root Mean Squared Error of Approximation (*RMSEA*), and the Standardized Root Mean Square Residual (*SRMR*) were used to assess goodness of fit^2–4^. Model fit was considered good when the CFI was higher than .95, the *RMSEA* was lower than .06, and the *SRMR* was lower than .08^5^. Cross-group invariance was tested comparing two nested models: the model of interest, in which no constraints were specified (i.e., the free model), was compared with a second – constrained – model where regression coefficients were constrained to be equal between groups (i.e., experimental conditions). Comparison of models was achieved using the nested robust χ^2^ test^6,7^: if a model comparison was statistically significant, the null hypothesis was rejected, meaning that the fit of a constrained model was worse than that of the free model^8^.

## Exploratory analyses

Several exploratory analyses were conducted on OT and subjective task importance. These analyses are reported below.

### Ongoing task

We analysed OT performance separately for average accuracy and reaction times (RTs) at correct OT trials. For both analyses, a 3 × 2 mixed ANOVA was used, with between-subjects factor *Condition* (control vs. single-cost vs. double-cost) and within-subjects factor *Task* (OT baseline – i.e., OT with no intention to be remembered – vs. TBPM). The results for OT accuracy showed that only the main effect of Condition was statistically significant, *F*(2, 203) = 5.47, *p* = .005, η²*_p_* = .05, whereas the main effect of Task (*p* = .674) and the interaction Condition × Task (*p* = .262) were not significant. Post-hoc Bonferroni comparisons indicated that, in both task blocks (OT baseline and TBPM) participants in the double-cost condition performed better than participants in the control, *t*(203) = 3.07, *p_adj_* = .007, and in the single-cost condition, *t*(203) = 2.58, *p_adj_* = .032; the difference between control and single-cost condition was not statistically significant (*p_adj_* = 1). The results for RTs at correct OT trials showed no significant effect for any of the two independent variables (*p* > .05). We further tested the PM cost in OT accuracy and in the RTs (in seconds) at the correct OT trials, separately. The PM cost was computed as difference (either in accuracy or in the RTs) between the OT baseline block (in which participants perform only the OT) to a TBPM block in which they perform the OT in the presence of a PM intention. The two one-way ANOVAs revealed that the Condition did not exert any significant effect on PM cost, neither in terms of OT accuracy (*p* = .262) nor in terms of RTs for correct OT trials (*p* = .946).

A series of partial correlations has been carried out to measure possible trade-off between OT and PM performance across experimental conditions. Correlation matrix is reported below; overall, only TBPM and OT accuracy positively correlated with each other (β = .16, *p* = .021), while all other correlations were not statistically significant (see Table 1).

### Subjective task importance

We explored the data on the subjective task importance, which was assessed by asking participants to respond to the following question. “During the task you just did, what was more important for you to carry out?”, and to indicate whether they perceived the OT or the TBPM task as more important, or whether both were perceived as equally important. We ran three exploratory analyses. In a first step, **a** χ^2^ **goodness-of-fit test** (χ^2^*_gof_*) was used to test whether the observed distribution of a subjective task importance differed across the three options (“OT”, “TBPM”, “both”). In the second step, we tested whether the distribution of choices of task importance differ across experimental conditions (control, single-cost, and double-cost) using **a Pearson’s** χ^2^ **test** (χ^2^*_Pearson_*). Finally, in the last step, we used a hierarchical multinomial logistic regression to investigate the effect of the experimental condition and behavioural performance (i.e., TBPM accuracy, absolute and relative clock-checking) on the perceived task importance.

The χ^2^ **goodness-of-fit test showed that participants chose the option “both” significantly more often (77%) than “TBPM” (17%) and “OT” (6%),** χ^2^*_gof_* (2) = 182.11, *p* < .001 (Figure 2, left panel)**; however, the Pearson’s** χ^2^ **test revealed that such choice distribution was not significantly affected by the experimental condition (*p* = .250;** Figure 2, right panel)**. The hierarchical multinomial logistic regression was carried out testing three models: in model 1, only TBPM accuracy was included as predictor; in model 2, both absolute and relative clock-checking and the correspondent interaction were included furtherly as predictors; in model 3, the experimental condition (control vs. single-cost vs. double-cost) was introduced as predictor. The reference level for the subjective task importance was set to “both”, whereas the reference level for the subjective task importance was set to “control”. Results indicated** that the fit between the model containing only the intercept and data improved with the addition of the TBPM accuracy in model 1, χ^2^ (2) = 6.49, *R^2^_Nagelkerke_* = .03, *p* = .039, of the time monitoring measures in model 2, χ^2^ (8) = 24.53, *R^2^_Nagelkerke_* = .11, *p* = .002, as well as of the experimental condition in model 3, χ^2^ (12) = 28.87, *R^2^_Nagelkerke_* = .13, *p* = .004. **However, model comparisons showed that model 2 – which included both measures of time monitoring and TBPM accuracy – fitted significantly better than model 1, which included only TBPM accuracy (**χ^2^(6) = 18.05, *p* = .006); however, adding the experimental condition as predictor in model 3 did not improve the model fit compared to model 2 (*p* = .362). Among the independent variables, only TBPM accuracy and absolute clock-checking predicted subjective task importance (Figure 3). Specifically, TBPM accuracy positively predicted the odds that participant choose the TBPM task as most important, *OR*: 5.85 (*95% C.I.*: 1.97, 17.41), *p* = .001, and negatively predicted the odds that participant choose the OT as most important task, *OR*: .03 (95% C.I.: .008, .091), *p* < .001 (Figure 3, left panel); absolute clock-checking negatively predicted the odds that participants choose the TBPM task as most important, *OR*: .90 (*95% C.I.*: .81, 1.00), *p* = .048 (Figure 3, right panel). All other effects were not statistically significant (*p* > .05). Overall, the analysis indicated that higher TBPM performance increased the probability of choosing the TBPM task as most important of ~485%, and decreased the probability of choosing the OT as most important of ~97.3%; moreover, higher absolute clock-checking reduced the probability of choosing the TBPM task as most important of ~9.70%.

# Questionnaires

In this study, several questionnaires have been administered. However, the data from these questionnaires were not analyzed in the main paper, as they are meant for future analyses.

## Perceived time passage

This was 5-points Likert scale that tested the subjective time experience of participants during both the OT and the time-based prospective memory (TBPM) task^9^. The questions were formulated for each of the two tasks separately, and were administered at the end of each task:

- *Question*: “During the task you just carried out, how fast did time pass for you?”
- *Likert scale*:
  1. *1*: “very slow”;
  2. *2*: “slow”;
  3. *3*: “neither fast nor slow”;
  4. *4*: “fast”;
  5. *5*: “very fast”.

## Loss aversion scale

This was a brief task that tested loss aversion^10^; participants indicated whether they accept or not the amount of an hypothetical money during a fictional head and tail coin game. Several scenarios of hypothetical bets were administered, and participants had to indicate whether they accept or reject each hypothetical bet. The scenarios were the following:

- *Scenarios*: “In the next slides, you will be asked whether you would accept or reject hypothetical coin flip bets. Please decide by your initial preference and don't think too long.”:
  1. “If the coin shows tails, you win 6 $, but if the coin is heads then you lose 3 $.”;
  2. “If the coin shows tails, you win 6 $, but if the coin is heads then you lose 2 $.”;
  3. “If the coin shows tails, you win 6 $, but if the coin is heads then you lose 4 $.”;
  4. “If the coin shows tails, you win 6 $, but if the coin is heads then you lose 5 $.”;
  5. “If the coin shows tails, you win 6 $, but if the coin is heads then you lose 6 $.”;
  6. “If the coin shows tails, you win 6 $, but if the coin is heads then you lose 7 $.”.
- *Response categories*:

1. *0*: “REJECT”;
   1. *1*: “ACCEPT”.

## Subjective time experience

This was a questionnaire that tested the subjective time experience ^10^ using a 5-point Likert scale. It assessed two mains constructs (personal time experience of present and past, and statements/metaphors on subjective time experience); both constructs comprise several sub-constructs, each of them assessed with few items as follows:

- *Construct 1 – Personal time experience of present and past*:
  1. *Sub-construct 1 – Personal time experience of present time*:
     1. “How fast does time usually pass for you?”;
     2. “How fast do you expect the next hour to pass?”.
  2. *Sub-construct 2 – Personal time experience of past time*:
     1. “How fast did the previous week pass for you?”;
     2. “How fast did the previous month pass for you?”;
     3. “How fast did the previous year pass for you?”;
     4. “How fast did the previous 10 years pass for you?”;
     5. “How fast did your childhood (before 12 years old) go by?”;
     6. “How fast did your youth (13-19 years old) go by?”.
  3. *Likert scale*:
     1. *1*: “very slow”;
     2. *2*: “slow”;
     3. *3*: “neither fast nor slow”;
     4. *4*: “fast”;
     5. *5*: “very fast”.
- *Construct 2 – Statements/metaphors on subjective time experience*:
  1. *Sub-construct 1 – Time pressure*:
     1. “I haven't enough time to complete my tasks.”;
     2. “I often feel time pressure.”;
     3. “I often haven't enough time to devote myself to important things.”;
     4. “I often think time is running out.”;
     5. “I have to establish my priorities, because I cannot do all the things I would like to do.”.
  2. *Sub-construct 2 – Time expansion*:
     1. “My time seems empty.”;
     2. “I often think that time just does not want to pass.”;
     3. “I often feel bored.”;
     4. “I have a lot of time.”;
     5. “I often have spent my time without doing anything.”.
  3. *Sub-construct 3 – Metaphors (time speed)*:
     1. “Time is a speeding train.”;
     2. “Time is a galloping horse.”;
     3. “Time is a tumbling waterfall.”.
  4. *Sub-construct 4 – Metaphors (time slowness)*:
     1. “Time is a vast expanse of sky.”;
     2. “Time is a quiet, motionless sea.”;
     3. “Time is a tedious song.”.
  5. *Likert scale*:
     1. *1*: “strong rejection”;
     2. *2*: “rejection”;
     3. *3*: “neutral”;
     4. *4*: “approval”;
     5. *5*: “strong approval”.

In the original questionnaire, there were two further questions for the *Sub-construct 2 – Personal time experience of past time* (i.e., “How fast did your adulthood between 20 and 29 years go by?”, and “How fast did your adulthood between 30 and 39 years go by?”), as this was a questionnaire originally thought to assess aging in time perception. Since we tested only younger adults, we removed these two questions as they were not pertinent for our sample.

## Follow-up questionnaire

This was a brief questionnaire that tested whether participants reported any strategy to track the passage of time during the TBPM task; specifically, participants were asked to give binary responses to this question, and only if they reported to have used a strategy, they were asked furtherly to provide a brief explanation, as follows:

- *Question 1*: “Did you use a strategy to control the passage of time during this task?”.
  1. *Response categories*:
     1. *0*: “NO”;
     2. *1*: “YES”.
- *Question 2* [only if participants indicated “YES”]: “What strategy did you use?”.

*Response*: written text.

# Tables & Figures

## Table 1

*Partial correlations*

|  |  | **TBPM accuracy** | | **Absolute clock-c.** | | **Relative clock-c.** | | **LDT accuracy** | | **OT accuracy** | | **LDT RTs** | | **OT RTs** | | **PM cost accuracy** | | **PM cost RTs** | |
| --- | --- | --- | --- | --- | --- | --- | --- | --- | --- | --- | --- | --- | --- | --- | --- | --- | --- | --- | --- |
| **TBPM accuracy** | *Pearson's r* | — |  |  |  |  |  |  |  |  |  |  |  |  |  |  |  |  |  |
|  | *p-value* | — |  |  |  |  |  |  |  |  |  |  |  |  |  |  |  |  |  |
| **Absolute clock-c.** | *Pearson's r* | 0.325 | *** | — |  |  |  |  |  |  |  |  |  |  |  |  |  |  |  |
|  | *p-value* | < .001 |  | — |  |  |  |  |  |  |  |  |  |  |  |  |  |  |  |
| **Relative clock-c.** | *Pearson's r* | 0.307 | *** | -0.3 | *** | — |  |  |  |  |  |  |  |  |  |  |  |  |  |
|  | *p-value* | < .001 |  | < .001 |  | — |  |  |  |  |  |  |  |  |  |  |  |  |  |
| **LDT accuracy** | *Pearson's r* | 0.116 |  | -0.067 |  | 0.027 |  | — |  |  |  |  |  |  |  |  |  |  |  |
|  | *p-value* | 0.096 |  | 0.338 |  | 0.705 |  | — |  |  |  |  |  |  |  |  |  |  |  |
| **OT accuracy** | *Pearson's r* | 0.161 | * | -0.053 |  | 0.044 |  | 0.811 | *** | — |  |  |  |  |  |  |  |  |  |
|  | *p-value* | 0.021 |  | 0.449 |  | 0.533 |  | < .001 |  | — |  |  |  |  |  |  |  |  |  |
| **LDT RTs** | *Pearson's r* | -0.023 |  | 0.027 |  | -0.097 |  | -0.122 |  | -0.15 | * | — |  |  |  |  |  |  |  |
|  | *p-value* | 0.747 |  | 0.696 |  | 0.174 |  | 0.082 |  | 0.032 |  | — |  |  |  |  |  |  |  |
| **OT RTs** | *Pearson's r* | 0.047 |  | 0.076 |  | -0.028 |  | -0.248 | *** | -0.254 | *** | 0.723 | *** | — |  |  |  |  |  |
|  | *p-value* | 0.5 |  | 0.276 |  | 0.692 |  | < .001 |  | < .001 |  | < .001 |  | — |  |  |  |  |  |
| **PM cost accuracy** | *Pearson's r* | -0.072 |  | -0.023 |  | -0.028 |  | 0.313 | *** | -0.301 | *** | 0.045 |  | 0.008 |  | — |  |  |  |
|  | *p-value* | 0.307 |  | 0.739 |  | 0.69 |  | < .001 |  | < .001 |  | 0.523 |  | 0.906 |  | — |  |  |  |
| **PM cost RTs** | *Pearson's r* | -0.093 |  | -0.061 |  | -0.097 |  | 0.151 | * | 0.12 |  | 0.451 | *** | -0.29 | *** | 0.051 |  | — |  |
|  | *p-value* | 0.186 |  | 0.388 |  | 0.171 |  | 0.03 |  | 0.086 |  | < .001 |  | < .001 |  | 0.463 |  | — |  |

*Note*. All correlations were controlled for the effect of the experimental condition (control, single-cost, double-cost). TBPM: time-based prospective memory; LDT: lexical decision task (i.e., ongoing task baseline); OT: ongoing task; clock-c.: clock-checking; RTs: reaction times (in seconds).

## Figure 1

*Raincloud plots of results from ANOVAs*

*
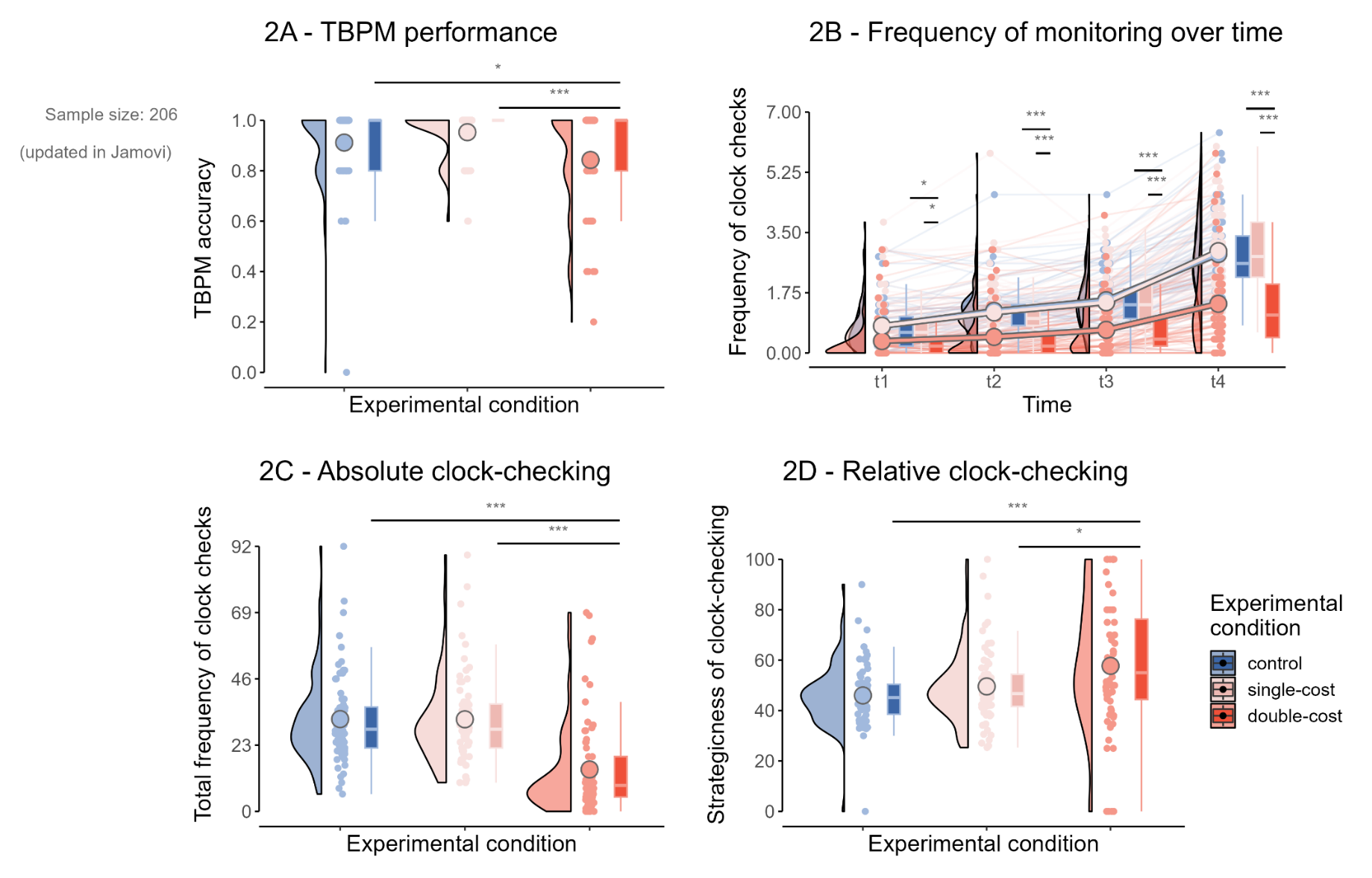
*

*Note.* The figure represents raincloud plots illustrating the distribution of data for the accuracy at the time-based prospective memory task (2A), the mean frequency of clock checks over time (2B), as well as absolute (2C) and relative clock-checking (as a percentage; 2D) as a function of the experimental conditions (monetary loss: control, single-loss, double-loss). Within each plot, the density of the data is represented on the left, individual data points are placed in the centre, and the boxplot is depicted on the right, separately for each experimental condition. The density plot provides insight into the overall group distribution, while the boxplot summarizes the central tendency and spread. TBPM: time-based prospective memory; t1: time 1 (i.e.: first 30 seconds’ interval before the PM target time); t2: time 2 (i.e.: second 30 seconds’ interval before the PM target time); t3: time 3 (i.e.: third 30 seconds’ interval before the PM target time); t4: time 4 (i.e.: fourth and last 30 seconds’ interval before the PM target time). * *p* < .05; ** *p* < .01; *** *p* < .001.

## Figure 2

*Subjective task importance and experimental manipulation*

| 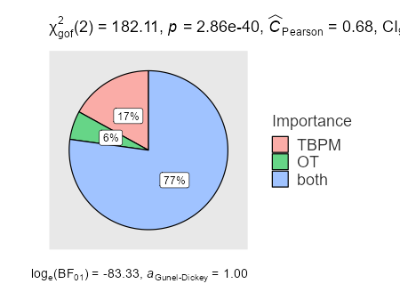 | 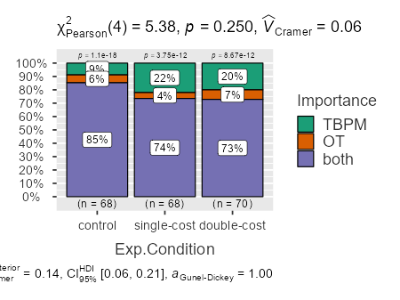 |
| --- | --- |

*Note.* The figure represents how choices of subjective task importance (both, OT, and TBPM) are distributed in the sample (left panel), and how they are distributed across experimental conditions (control, single-cost, double-cost). TBPM: time-based prospective memory; OT: ongoing task.

## Figure 3

*Main results from hierarchical multinomial logistic regression*

| *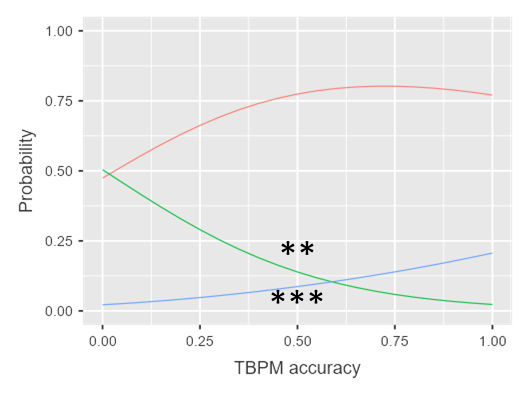* | *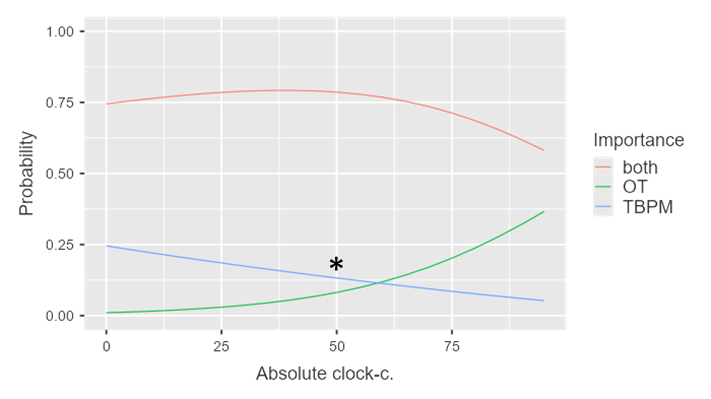* |
| --- | --- |

*Note.* The figure represents probabilities of subjective task importance (both, OT, and TBPM) as a function of the time-based prospective memory accuracy (left panel) and absolute clock-checking (right panel). TBPM: time-based prospective memory; OT: ongoing task; Absolute clock-c.: absolute clock-checking. * *p* < .05; ** *p* < .01; *** *p* < .001.

# References

1. Carlson, H. A. Check Your Confidence: Size Really *Does* Matter. *J. Chem. Inf. Model.* **53**, 1837–1841 (2013).

2. Bentler, P. M. Quantitative methods in psychology: Comparative fit indexes in structural models. *Psychol. Bull.* **107**, 238–246 (1990).

3. Hu, L. T. & Bentler, P. M. Cutoff criteria for fit indexes in covariance structure analysis: Conventional criteria versus new alternatives. *Struct. Equ. Model.* **6**, 1–55 (1999).

4. Steiger, J. H. Structural Model Evaluation and Modification: An Interval Estimation Approach. *Multivar. Behav. Res.* **25**, 173–180 (1990).

5. Hooper, D., Coughlan, J. & Mullen, M. R. Structural Equation Modelling: Guidelines for Determining Model Fit. *Electron. J. Bus. Res. Methods* **6**, 53–60 (2008).

6. Bentler, P. M. & Satorra, A. Testing model nesting and equivalence. *Psychol. Methods* **15**, 111–123 (2010).

7. Pavlov, Y. G. & Kotchoubey, B. Oscillatory brain activity and maintenance of verbal and visual working memory: A systematic review. *Psychophysiology* **59**, (2020).

8. Chen, F. F. Sensitivity of Goodness of Fit Indexes to Lack of Measurement Invariance. *Struct. Equ. Model. Multidiscip. J.* **14**, 464–504 (2007).

9. Thönes, S. & Stocker, K. A standard conceptual framework for the study of subjective time. *Conscious. Cogn.* **71**, 114–122 (2019).

10. Gächter, S., Johnson, E. J. & Herrmann, A. Individual-level loss aversion in riskless and risky choices. *Theory Decis.* **92**, 599–624 (2022).
